# The Psychedelic State Induced by Ayahuasca Modulates the Activity and Connectivity of the Default Mode Network

**DOI:** 10.1371/journal.pone.0118143

**Published:** 2015-02-18

**Authors:** Fernanda Palhano-Fontes, Katia C. Andrade, Luis F. Tofoli, Antonio C. Santos, Jose Alexandre S. Crippa, Jaime E. C. Hallak, Sidarta Ribeiro, Draulio B. de Araujo

**Affiliations:** 1 Brain Institute, Federal University of Rio Grande do Norte (UFRN), Natal-RN, Brazil; 2 Department of Medical Psychology and Psychiatry, University of Campinas (UNICAMP), Campinas-SP, Brazil; 3 Department of Neuroscience and Behavior, University of Sao Paulo (USP), Ribeirao Preto-SP, Brazil; College of Mechatronics and Automation, National University of Defense Technology, CHINA

## Abstract

The experiences induced by psychedelics share a wide variety of subjective features, related to the complex changes in perception and cognition induced by this class of drugs. A remarkable increase in introspection is at the core of these altered states of consciousness. Self-oriented mental activity has been consistently linked to the Default Mode Network (DMN), a set of brain regions more active during rest than during the execution of a goal-directed task. Here we used fMRI technique to inspect the DMN during the psychedelic state induced by Ayahuasca in ten experienced subjects. Ayahuasca is a potion traditionally used by Amazonian Amerindians composed by a mixture of compounds that increase monoaminergic transmission. In particular, we examined whether Ayahuasca changes the activity and connectivity of the DMN and the connection between the DMN and the task-positive network (TPN). Ayahuasca caused a significant decrease in activity through most parts of the DMN, including its most consistent hubs: the Posterior Cingulate Cortex (PCC)/Precuneus and the medial Prefrontal Cortex (mPFC). Functional connectivity within the PCC/Precuneus decreased after Ayahuasca intake. No significant change was observed in the DMN-TPN orthogonality. Altogether, our results support the notion that the altered state of consciousness induced by Ayahuasca, like those induced by psilocybin (another serotonergic psychedelic), meditation and sleep, is linked to the modulation of the activity and the connectivity of the DMN.

## Introduction

The overwhelming experience induced by psychedelics such as LSD, psilocybin, mescaline and N,N-dimethyltryptamine (DMT) share common characteristics [1]. From the neuropsychological perspective, they are all substances known to induce complex mystical experiences, modulating the sensory, perceptual, cognitive and autonomic systems, as well as emotional processes. A renowned effect of these substances is the increased vividness of both perception and internally generated mental images, therefore the name hallucinogens [1,2].

In order to gain insight into the neurophysiology underlying mental imagery induced by psychedelics, we have used functional Magnetic Resonance Imaging (fMRI) to investigate the effects of Ayahuasca, a powerful psychedelic traditionally used by Amazonian Amerindians, and then spread to Western countries, occupying the core of some Brazilian syncretic religions [3,4]. This beverage is produced from the decoction of two different plants: the *Psychotria viridis* and the *Banisteriopsis caapi*. The first one contains the psychedelic tryptamine N,N-dimethyltryptamine (DMT) that binds to serotonin and sigma-1 receptors [4,5]. The second is rich in beta-carboline alkaloids, particularly harmine, tetrahydroharmine (THH), and harmaline. Harmine and harmaline are potent monoamine oxidase inhibitors (MAOi) and THH acts as a mild selective serotonin reuptake inhibitor and a weak MAOi [6]. During execution of a mental imagery task, we found that Ayahuasca selectively modulates frontal, temporal and occipital brain networks associated with intention, memory and vision [7]. The effects include significant increased activity of the primary visual cortex, in tight correlation with psychometric changes [7].

In addition to the amplification of mental imagery, the experience induced by Ayahuasca involves sedation, gastrointestinal distress, changes of spatiotemporal scaling, dissociation, sense of well-being, insights, feelings of apprehension, and, very notably, increased introspection (the inspection of one owns thoughts and feelings) [2,8–11]. A group of brain regions that has lately received considerable attention due to its consistent association with internally oriented mental processes is the Default Mode Network (DMN) [12–17]. This network presents increased activity while one is at rest, in comparison with activity levels detected during execution of externally oriented cognitive task [14,18]. Accordingly, mind wandering, a private, continuous and often unnoticed phenomenon, is known to involve the DMN [13,19,20]. Indeed, functional neuroimaging studies have consistently associated the activity of the DMN with key components of mind-wandering, such as remembering the past and planning the future [21,22].

The activity of the DMN is sensitive to a number of tasks, states of consciousness and psychiatric conditions. On one end of the spectrum, DMN activity is increased in schizophrenia [23], depression [12], Parkinson’s disease [24], social phobia [25], and by tetrahydrocannabinol administration (THC) [26]. On the other end, DMN activity is reduced in autism [27], Alzheimer's disease [28], during hypnosis [29], meditative states [17], and also by psilocybin intake [16].

Resting state fMRI (rs-fMRI) has allowed the evaluation of how DMN areas interact with each other, for instance by means of functional connectivity (fc-fMRI) measurements [30]. Indeed, this technique has proven to be sensitive enough to detect connectivity changes between DMN nodes associated with a number of altered states of consciousness, such as sedation, sleep, meditation, and psilocybin [31–35]. Furthermore, rs-fMRI has lead to the suggestion that the DMN exhibits an intrinsic competitive behavior with another brain network related to externally oriented tasks, known as task-positive network (TPN) [36]. The anti-correlation between DMN and TPN found in rs-fMRI indicates that when the signal of one network is increased, the signal of the other tends to decrease [36,37].

Although Ayahuasca intake increases introspection [11], it is expected that it also reduces DMN activity, analogous to psilocybin and meditation. Hence, the first aim of this study was to investigate how Ayahuasca interferes with the DMN activity, using an active task. We also used rs-fMRI to investigate how Ayahuasca experience interferes with functional connectivity between different DMN regions. Lastly, we analyzed the impact of Ayahuasca over the TPN-DMN orthogonality, to assess whether part of the Ayahuasca experience comes from an increased overlap between internally and externally oriented attention.

## Materials and Methods

### Ethics Statement

The work was approved by the Ethics and Research Committee of the University of Sao Paulo at Ribeirao Preto (N° 14672/2006), and written informed consent was obtained from all subjects that participated in this study.

### Subjects

Ten healthy volunteers (5 males; mean age of 29 years, ranging from 24 to 48 years) with at least 5 years of regular (twice a month) Ayahuasca use were recruited from Santo Daime church to participate in this study. They were all right-handed, had no comorbid history of neurological or psychiatric disorder as assessed by DSM-IV structured interview, and were not under medication for at least 3 months prior to the study. Subjects were abstinent from caffeine, nicotine and alcohol prior to scanning session. Data from one volunteer was excluded from analyses due to excessive head movement. The final dataset included 9 subjects (five women). All presented limited motion artifact: before (0.14 ± 0.05 mm), and after Ayahuasca intake (0.09 ± 0.11 mm), which were not significantly different (Wilcoxon paired test—p-value = 0.13). An additional group of 26 control individuals (11 males; mean age: 37.39 years) also participated in this study in order to define the Regions of Interest (ROI) of the DMN.

### Experimental design

Subjects underwent two fMRI sessions, before and 40 min after Ayahuasca intake (2.2 mL/kg of body weight). The Ayahuasca used was provided by the Santo Daime church and contained 0.8 mg/ml of DMT and 0.21 mg/ml of harmine. Presence of harmaline was not detect under the chromatography threshold of 0.02 mg/mL [7]. In each session subjects performed two protocols. The first one was used to evaluate DMN activity. It consisted of a verbal fluency task, designed as a block paradigm, which alternated six blocks of rest with five blocks of silently generated words starting with a cued letter (M, A, E, C and S, one letter for each block). Each block lasted 27.5 seconds. During rest periods the subjects were asked to think of a white wall. The second protocol was a conventional rs-fMRI acquisition, designed to evaluate the functional connectivity of the DMN. During acquisition, subjects were asked to keep their eyes closed and to hold their head as still as possible. To evaluate subjective effects caused by Ayahuasca, two psychiatric scales were applied at time 0 (baseline), 40 minutes, 80 minutes and 200 minutes after Ayahuasca ingestion: the Brief Psychiatric Rating Scale (BPRS), and the Young Mania Rating Scale (YMRS) [7].

### Image acquisition

Images were acquired in a 1.5 T scanner (Siemens, Magneton Vision). For the verbal fluency task, a total of 66 volumes were acquired, each with 16 slices, using an EPI sequence with the following parameters: TR = 4600 ms; TE = 66 ms; FOV = 220 mm; matrix 128 x 128; voxel dimensions of 1.72 mm x 1.72 mm x 5.00 mm. For the resting state protocol, 150 volumes were acquired with the same parameters used in the block paradigm except for the TR, which was set to 1700 ms, and a matrix of 64 x 64. Whole brain high resolution T1-weighted images were also acquired, consisting of 156 contiguous sagittal slices using a multiplanar reconstructed gradient-echo sequence, with the following parameters: TR = 9.7ms; TE = 44ms; flip angle 12°; matrix 256 x 256; FOV = 256 mm, voxel size = 1 mm x 1 mm x 1 mm.

### fMRI pre-processing

Preprocessing of the functional dataset was performed in FSL software (Analysis Group FRMIB, Oxford, UK), and included slice-timing correction, head motion correction, and spatial smoothing (Gaussian kernel, FWHM = 5 mm). EPI were first registered to the anatomical images using a rigid body transformation (6 DOF), and then normalized to the Montreal Neurologic Institute (MNI) 152 template using a linear transformation (12 DOF).

### DMN ROI definition

To avoid double dipping, i.e., the use of the same data set for selection and selective analysis [38], the DMN Regions of Interest (ROI) were defined based on data from the control group, who performed the same verbal fluency task. Statistical analysis was conducted in SPM5 (Statistical Parametric Mapping, Wellcome Trust Centre for Neuroimaging, London, UK). For both control and experimental groups, fMRI time courses were high-pass filtered at 0.015 Hz and analyzed using the General Linear Model (GLM) with a regressor modeling the task periods, convolved with a canonical hemodynamic response function (HRF). For each subject, a contrast was applied (rest > task) using t-test. The contrasts then entered into a second level random effect analysis to create statistical maps from the groups of subjects (q[FDR] < 0.01, cluster size of 50 contiguous voxels).

The DMN mask used was based on the intersection of the statistically significant voxels with the following nine brain areas consistently associated with the DMN [39]: the anterior cingulate cortex (ACC), the posterior cingulate cortex (PCC), the precuneus (PC), the medial prefrontal cortex (mPFC), the left middle frontal gyrus (LMFG), the left and right middle temporal gyrus (LMTG and RMTG), and the left and right inferior parietal lobule (LIPL and RIPL).

### Effects of Ayahuasca over the DMN

Experimental group maps were compared between conditions. For each subject, a contrast was applied (rest > task) using a t-test. The contrasts then entered into a second level fixed effect analysis in order to perform a paired comparison of the two instants: before (DMN-Before) and after (DMN-After). Statistical maps presented onto inflated cortical surfaces were constructed using the FreeSurfer package (version 5.0.0, http://surfer.nmr.harvard.edu). Furthermore, averaged β-values and standard deviations were extracted from the nine ROI in the experimental group, both before and after Ayahuasca intake. Post-hoc statistical analyses were performed in GraphPad Prism 4.00 (GraphPad Software, San Diego, CA, EUA), using the Wilcoxon paired test, corrected for multiple comparisons by the total number of ROI (nine).

Mean individual β-values were also extracted for each ROI, before and after Ayahuasca ingestion. They were used to compute the correlation between individual β-values change (Δβ = β_after_—β_before_) and the individual scores of the psychometric scales at 80 min (right after MRI scanning), corrected for multiple comparisons by 12 (6 ROI x 2 scales).

### fMRI functional connectivity (fc-fMRI)

Images from the resting state paradigm were used to evaluate seed-based functional connectivity (fc-fMRI), in the software REST (Resting-state fMRI Data Analysis Toolkit, version 1.7 [40]). To reduce spurious correlations, averaged time courses from white matter, cerebrospinal fluid, global signal, and the six rigid-body realignment parameters were regressed out using SPM. The residual time series were then filtered between 0.01 and 0.08 Hz. One seed (10 mm radius sphere) was defined at the two main hubs of the DMN: PCC (MNI coordinates: -4, -47, 45) and mPFC (MNI coordinates: 0, 51, -14), according to [36]. Average time series extracted from these two seeds (both before and after Ayahuasca) served to calculate their temporal correlations with every other voxel in the brain. Results were expressed in terms of voxel-wise Pearson coefficients, z-Fisher transformed. A group analysis was performed concatenating the z-maps of both seeds and comparing the sessions (before and after Ayahuasca) through a paired t-test. Additionally, statistical differences were assessed considering the z-maps of each seed separately. Statistical differences were evaluated only in the nine ROI previously defined.

### DMN x TPN orthogonality

Herein we used a classical approach to define the TPN [36], where three seed regions (10 mm radius spheres) are selected, each one centered at: intraparietal sulcus—IPS (MNI coordinates: -25, -54, 53), frontal eye field—FEF (MNI coordinates: 29,-7, 52) and middle temporal region—MT (MNI coordinates: -47, -72, 1). The same filtered residual time series from the fc-fMRI analysis were used to construct the correlation maps. Before Ayahuasca intake, correlations maps were built, one for each seed, and Fisher’s z-transformed. The z-maps of the three seeds entered into a one-sample t-test, and then binarized to obtain a TPN mask using a threshold of p < 0.001. DMN was defined based on PCC and mPFC seeds as described in the fc-fMRI section. This mask and the DMN mask were used to extract average time series for each subject, from which Pearson’s correlation coefficients were calculated between DMN and TPN for both conditions (before and after) separately. Statistical differences were assessed using a paired t-test, and threshold was set at p < 0.05. Moreover, as it has been suggested that global signal regression may create false negative correlations [41,42], data was also analyzed keeping the global signal in the pre-processing step.

## Results

**Fig 1:**
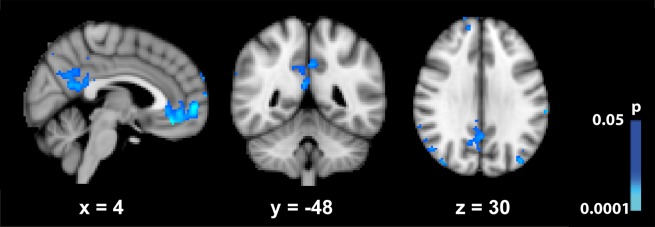
Statistical maps showing regions where BOLD signal of the DMN (rest > task) decreases after Ayahuasca ingestion. P < 0.05 uncorrected.

The mask that defined the DMN, outlined from a control group, is shown in S1 Fig. Based on these ROI, the signal of the DMN changed significantly when comparing rest with task periods, both before and after Ayahuasca intake (S2 Fig.). Statistical comparison between maps obtained for the two moments (before vs. after) revealed a significant signal decrease for most parts of the DMN as a result of Ayahuasca intake (Fig. 1). Two ROIs (LMFG and LMTG) presented a significant signal increase after Ayahuasca intake. These structures are known to be involved in language processing (Binder, et al., 1997), and since we used a verbal fluency task it is possible that they are also engaged during task execution, therefore confounding the interpretation of these results.

A further ROI inspection confirmed that Ayahuasca effects are accompanied by statistically significant reduction of activity for most parts of the DMN (p < 0.01, Bonferroni corrected for multiple comparisons). S3 Fig. shows the average β-values and standard error for both sessions (before and after Ayahuasca ingestion), in each of the 9 ROI. Significant decreased β-values were observed in ACC, PCC, MPFC, PC and bilateral IPL (S3 Fig., Table 1).

**Table 1:** Changes in DMN (rest > task) BOLD signal following Ayahuasca intake.

		**β-values Before Ayahuasca**		**β-values After Ayahuasca**			
**ROI**	**Nvoxel**	**Mean**	**SD**	**Mean**	**SD**	**P value**	
ACC	606	0.4193	0.2797	0.3188	0.1497	< 0.0001	*
PCC	2058	0.6409	0.3636	0.5378	0.2397	< 0.0001	*
MPFC	2963	0.551	0.3701	0.4211	0.2854	< 0.0001	*
RIPL	3216	0.5226	0.2997	0.4620	0.2612	< 0.0001	*
LIPL	1749	0.4464	0.2650	0.3795	0.2392	< 0.0001	*
LMFG	98	0.0798	0.0830	0.2140	0.1419	< 0.0001	*
RMTG	1731	0.1858	0.1631	0.1860	0.1309	0.7996	
LMTG	1028	-0.0211	0.1868	0.0651	0.1740	< 0.0001	*
PC	3929	0.8131	0.3491	0.7818	0.3482	< 0.0001	*

Nvoxel = number of voxels in each ROI, mean and standard deviation of β-values before and after Ayahuasca ingestion (in % BOLD signal change). * Indicates significant differences after Ayahuasca (p<0.0011, corresponding to p<0.01 corrected for multiple comparisons by the number of ROI).

The correlation analysis between individual Δβ and corresponding psychiatric scales revealed a tendency for significant negative correlations between YMRS scores 80 minutes after Ayahuasca ingestion only at ACC (r = -0.78, p = 0.072, Bonferroni corrected for 12).

**Fig 2:**
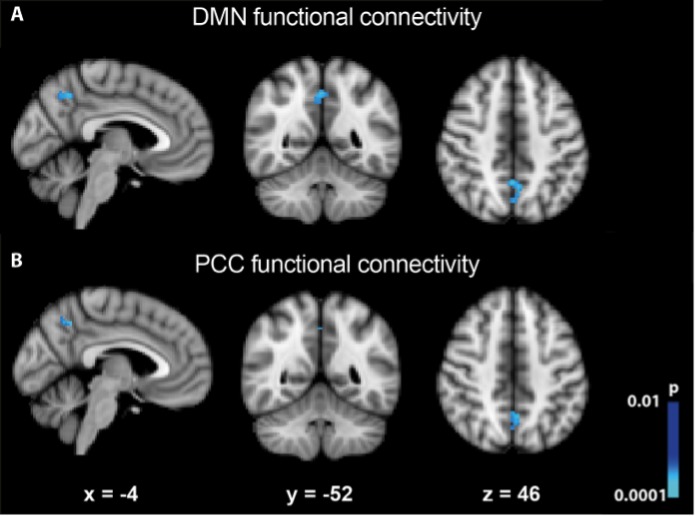
Changes in functional connectivity of DMN. (A) Connectivity within the PCC/Precuneus decreased after Ayahuasca ingestion. (B) Considering only the PCC seed, we can observe that this seed drives the contribution for the decrease in DMN connectivity. Images were thresholded using a cluster corrected pcluster < 0.01 (using a voxel collection threshold of p < 0.001).

To inspect how the different regions of the DMN relate to each other, fc-fMRI maps were constructed before and after Ayahuasca ingestion (S4 Fig.). The direct statistical comparison of these maps exhibited a significant functional connectivity decrease within the PCC/Precuneus (Fig. 2A). By analyzing each seed independently, it appears that most of the contribution for the observed connectivity decrease is driven by the PCC (Fig. 2B). We did not observe significant connectivity changes induced by Ayahuasca when explicitly evaluating the correlation between mPFC and PCC seeds (r^2^ = 0.13; -0.02, before and after Ayahuasca ingestion respectively, p = 0.12).

**Fig 3:**
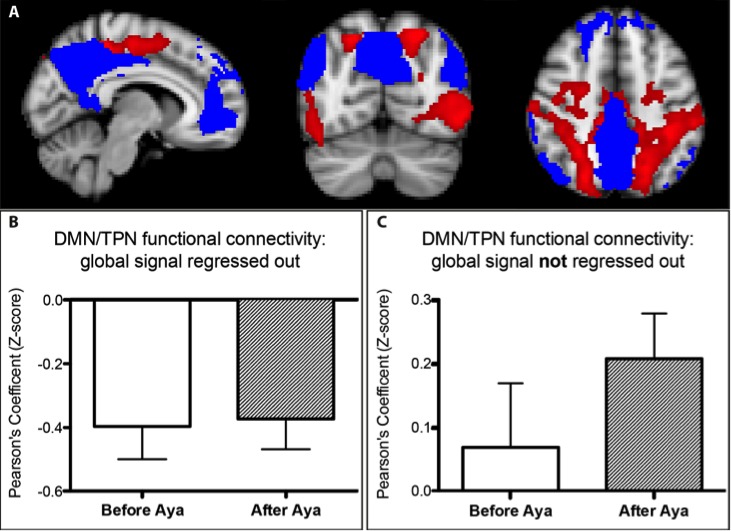
Task Positive and Default Mode Networks. (A) TPN (red) and DMN (blue) masks are shown. (B) TPN and DMN were anti-correlated when the global signal was regressed out, no significant alterations are observed following Ayahuasca intake. (C) Without regression against global signal, TPN and DMN were positively correlated and no significant changes were observed after Ayahuasca ingestion.

In addition to investigating the changes in the activity and connectivity of the DMN, we also analyzed its interaction with the TPN. In the first analysis, following most studies that compute DMN-TPN correlations, the global signal was regressed out during data pre-processing. Results are presented in Fig. 3. Fig. 3A shows the masks used for TPN (red) and DMN (blue). Fig. 3B shows the expected anti-correlation between TPN and DMN. There was no statistically significant difference in DMN-TPN orthogonality when comparing before and after Ayahuasca ingestion (Fig. 3B).

As increasing evidences have been pointing out to the fact that regression against global signal induces spurious negative correlation [41–43], we re-analyzed this dataset without global signal regression. In this new configuration, the DMN-TPN connectivity pattern presented a positive correlation, both before and after Ayahuasca intake. Although there is a trend of increased positive correlation, again no significant difference was found in the DMN-TPN relationship after Ayahuasca intake (p = 0.22) (Fig. 3C).

## Discussion

Our data demonstrate that Ayahuasca intake leads to a decrease in the activity of core DMN structures.

There are at least two not mutually excluding hypotheses to explain these results. The first one relates to the very definition of the DMN as a network that disengages when individuals are performing a cognitive task. It is possible that the decrease in DMN activity after the intake of psychedelics comes from the level of concentration and mind effort demanded during the Ayahuasca experience [1]. For instance, religious and shamanic traditions refer to the Ayahuasca experience as “labor”. Therefore, it is likely that resting subjects under the influence of psychedelics deal with the experience in a very individual manner, effectively performing internally-generated “labor” equivalent to a task, especially if we consider that the subjects investigated were all experienced users [16].

The second hypothesis stems from the consistent evidence that DMN activity is also reduced during meditative states [13,17,44]. In fact, psychedelics and meditation share many psychological features. For instance, both conditions increase introspection, self-perception, and affect mind-wandering [1,11,45]. Meditation has been described as “the *practice* of resting your attention on whatever is going through your mind, without the attempt of interfering or attaching to the stream of thoughts and emotions” [46]. Decreased DMN activity during meditation has been linked to a decrease in mind-wandering [17]. This should not be the case with psychedelics, as experienced users show potentiated mind-wandering [16]. On the other hand, the awareness of mind-wandering is altered in both states. A recent study suggests that DMN activity increases during periods of mind-wandering, but decreases with the awareness that the mind has wandered [47], and this could be just the case of the Ayahuasca experience. It is as if these experiences lead to a change of standpoint, shifting one’s perspective from actor to an attentive spectator.

Although there are similarities, each experience has its own particularities. In fact, this is reflected by our own fc-fMRI results, in particular the observed reduction of functional connectivity within the PCC/Precuneus, while the mPFC-PCC functional connectivity was not significantly altered. In its turn, meditation has been consistently associated with stronger coupling between anterior and posterior nodes of the DMN [17,31,48].

Lately, fc-fMRI has also served as potential marker of different altered states of consciousness, particularly due to changes over posterior midline nodes of the DMN, such as the PCC/Precuneus. For instance, transition from wakefulness to the N1 stage of sleep is marked by decreased connectivity of the PCC with the rest of the DMN, while mPFC connectivity exhibits a stepwise decrease as sleep depth increases [15]. An analogous reduction in PCC connectivity is observed during light sedation with midazolam [32].

Although overall similar to the changes observed for psilocybin, the changes induced by Ayahuasca did not find a significant reduced coupling between PCC and mPFC, as observed after psilocybin intake [16]. Again, although Ayahuasca and psilocybin have much in common, the uniqueness of the experience brought by each substance should be remarked. The Ayahuasca experience usually involves much stronger somatic and sedation effects. From the neuropharmacology perspective, psilocybin acts almost exclusively on the serotonergic system, while Ayahuasca is linked to a rich combination of neurochemical mechanisms: DMT is a trace amine with affinities to sigma-1, monoaminergic, and trace amine-associated receptors [5,8,49,50]. Furthermore, Ayahuasca contains inhibitors of mono-amino-oxidases, which prevents the degradation of monoamine neurotransmitters and thus increase their levels.

DMN signal reduction in the ACC showed a tendency of negative correlation after correction for multiple comparison (p < 0.072) with individual YMRS scores. This scale is designed to access changes on mood, sleep, irritability, speech, language-thought, among other manic symptoms [51]. The impact of Ayahuasca on the YMRS scores were mainly influenced by mood, affect, speech, and content of thoughts, and did not indicate a more generalized mania-like state (S5 Fig.). This finding suggests that the subjective changes caused by Ayahuasca, particularly in the content and structure of thoughts, is associated with the modulation of the anterior part of the DMN.

In addition to investigating the changes in the activity and connectivity of the DMN, we also examined its interaction with the TPN. When the global signal was regressed out, the DMN was anti-correlated to the TPN (Fig. 3B), as expected [36]. Such anti-correlation pattern did not change significantly after Ayahuasca intake. This contrasts with evidence that altered states of consciousness produced by propofol [52], psilocybin [35] and meditation [53] involve a substantial impact on the orthogonality of these networks. It is however worth noting that some of these studies included correction for global signal fluctuations, and this has been lately pointed out as a potential problem when inspecting negative correlations from rs-fMRI. To address this problem, we re-inspected our data keeping the global signal. A positive DMN-TPN correlation emerged, but again no significant correlation change was found after Ayahuasca intake (Fig. 3C). The validity of regressing out the global signal to evaluate DMN-TPN orthogonality is still a matter of debate [41–43,54]. In fact, there is a mounting body of evidence suggesting that the intrinsic DMN-TPN anti-correlation may result from artifacts introduced by regression against global signal [41,42], as we have observed.

The present study concludes that the acute effects of Ayahuasca are associated with diminished DMN activation and decreased functional connectivity of the PCC/Precuneus. Altogether, our results support the notion that the altered state of consciousness induced by Ayahuasca, like with psilocybin, meditation and sleep, is linked to the modulation of the activity and the connectivity of the DMN.

### Limitations

This study has some caveats and limitations. First, we limited the study to experienced users. This decision was made to avoid vomiting and diarrhea inside the scanner, two effects frequently observed in naïve subjects. Therefore, the current data cannot clarify whether the effects observed were solely due to the chemical properties of Ayahuasca, or whether previous experience also played a significant role. Second, we did not include a control group treated with a placebo, because it is difficult if not impossible to develop a placebo credible for experienced users, i.e. a substance void of psychoactive chemicals but able to match Ayahuasca’s strong and particular flavor. However, it is worth nothing that a number of other studies have already demonstrated that the robust psychological, electrophysiological and neuroimaging [8,11] changes induced by Ayahuasca intake are not explained by a typical placebo effect. The fact that the scans took place inside a hospital, away from the traditional setting of Ayahuasca intake for experienced subjects, also weakens the placebo hypothesis. Third, fixed effects analysis was used, which may limit the results to the group of subjects studied. Forth, the acquisition of fMRI signal was not controlled for order effects. Again, the magnitude of the observed changes is very robust, encompassing six hubs of the DMN. Furthermore, the two sessions were both in the same day, only two hours apart. This protocol guarantees that a stable mental condition of the subjects in both sessions and naturally forbids the control for order, especially for a substance with such strong effects. Unraveling the contributions of chemistry, belief and ritual setting for the hallucinogenic effects of Ayahuasca will require further experimentation. Future studies with larger samples and comparing long-term versus occasional Ayahuasca users are also desired and opportune.

## Supporting Information

S1 Fig DMN mask. This mask was obtained from a control group (26 individuals) that performed the same verbal fluency task as the experimental group. Images were thresholded at q[FDR] < 0.01, cluster size of 50 contiguous voxels.
(TIF)

S2 Fig DMN maps before and after Ayahuasca intake. Images were thresholded using cluster corrected pcluster < 0.05 (using a voxel collection threshold of p < 0.001).
(TIF)

S3 Fig Mean β-values from ROIs of the DMN. Bar plots of the mean β-values from ROIs of the DMN where BOLD signal was altered after Ayahuasca ingestion. Decreases were found in most of ROI: ACC, MPFC, PCC, PC and bilateral IPL. *p < 0.01 correct for multiple comparisons by the number of ROI (nine).
(TIF)

S4 Fig Functional connectivity maps obtained for the PCC and mPFC seeds independently. Maps are shown at the two moments: before (left side) and after (right side) Ayahuasca ingestion. The resulting statistical maps were thresholded at p < 0.05 uncorrected.
(TIF)

S5 Fig YMRS changes. Only 5 out of the 11 YMRS items showed statistically significant changes after Ayahuasca intake. Bars present score values (mean + SE). P < 0.05 uncorrected.
(TIF)
