# Supplementary figures and images for: The Psychedelic State Induced by Ayahuasca Modulates the Activity and Connectivity of the Default Mode Network
Source: PLoS One. 2015 Feb 18;10(2):e0118143. doi: 10.1371/journal.pone.0118143 (PMC4334486; doi:10.1371/journal.pone.0118143)

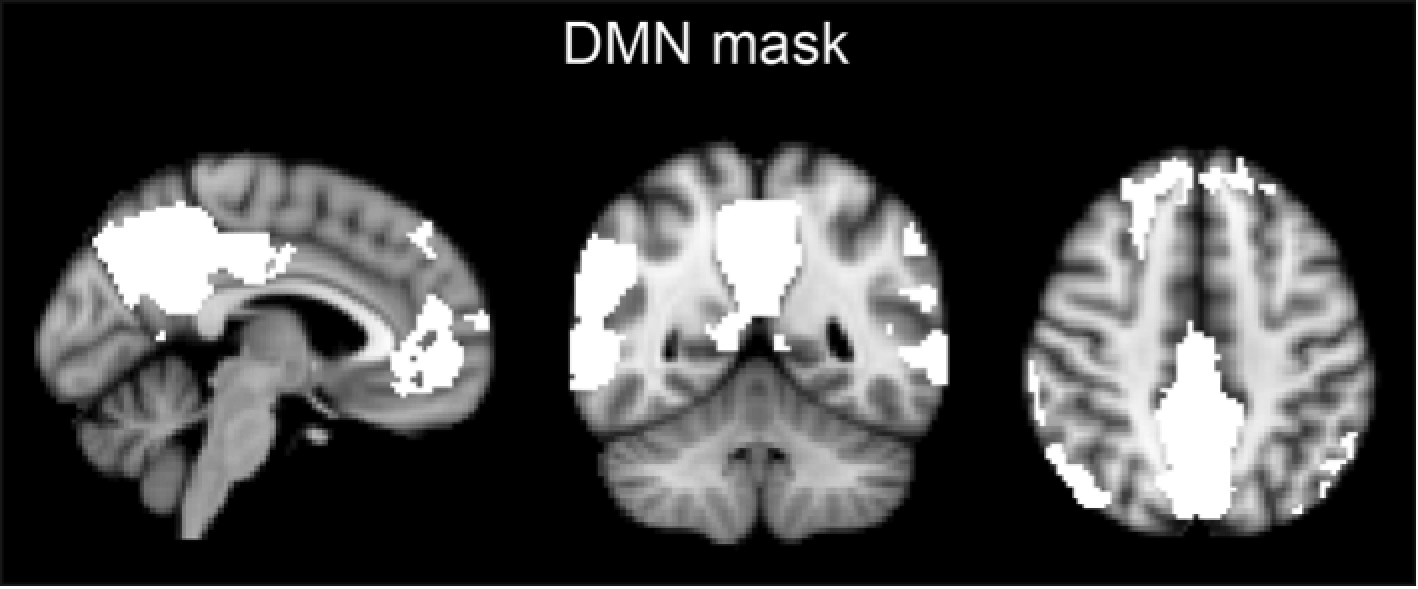

Supplement: S1 Fig — This mask was obtained from a control group (26 individuals) that performed the same verbal fluency task as the experimental group. Images were thresholded at q[FDR] < 0.01, cluster size of 50 contiguous voxels. (TIF) [file pone.0118143.s001.tif]

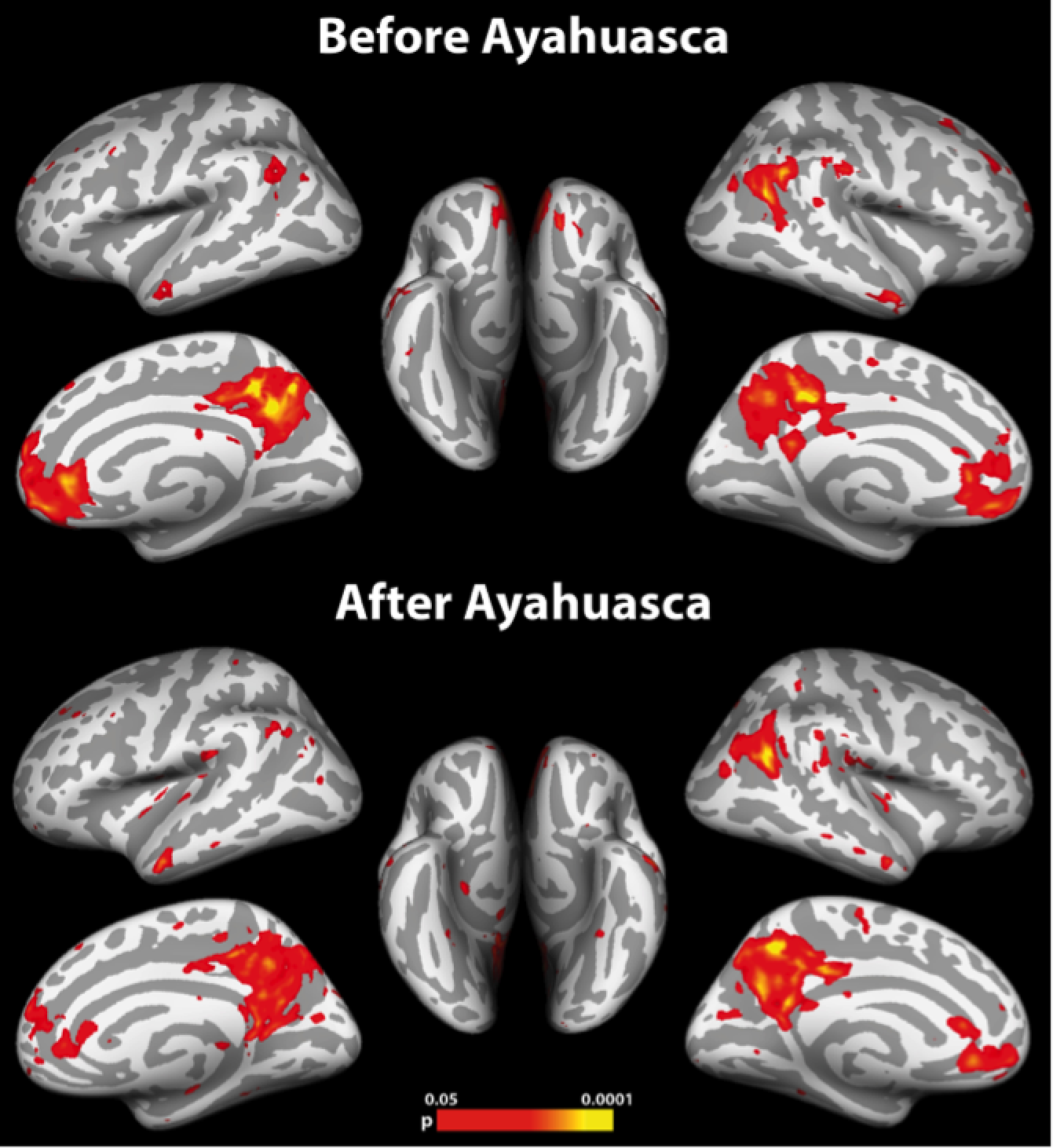

Supplement: S2 Fig — Images were thresholded using cluster corrected pcluster < 0.05 (using a voxel collection threshold of p < 0.001). (TIF) [file pone.0118143.s002.tif]

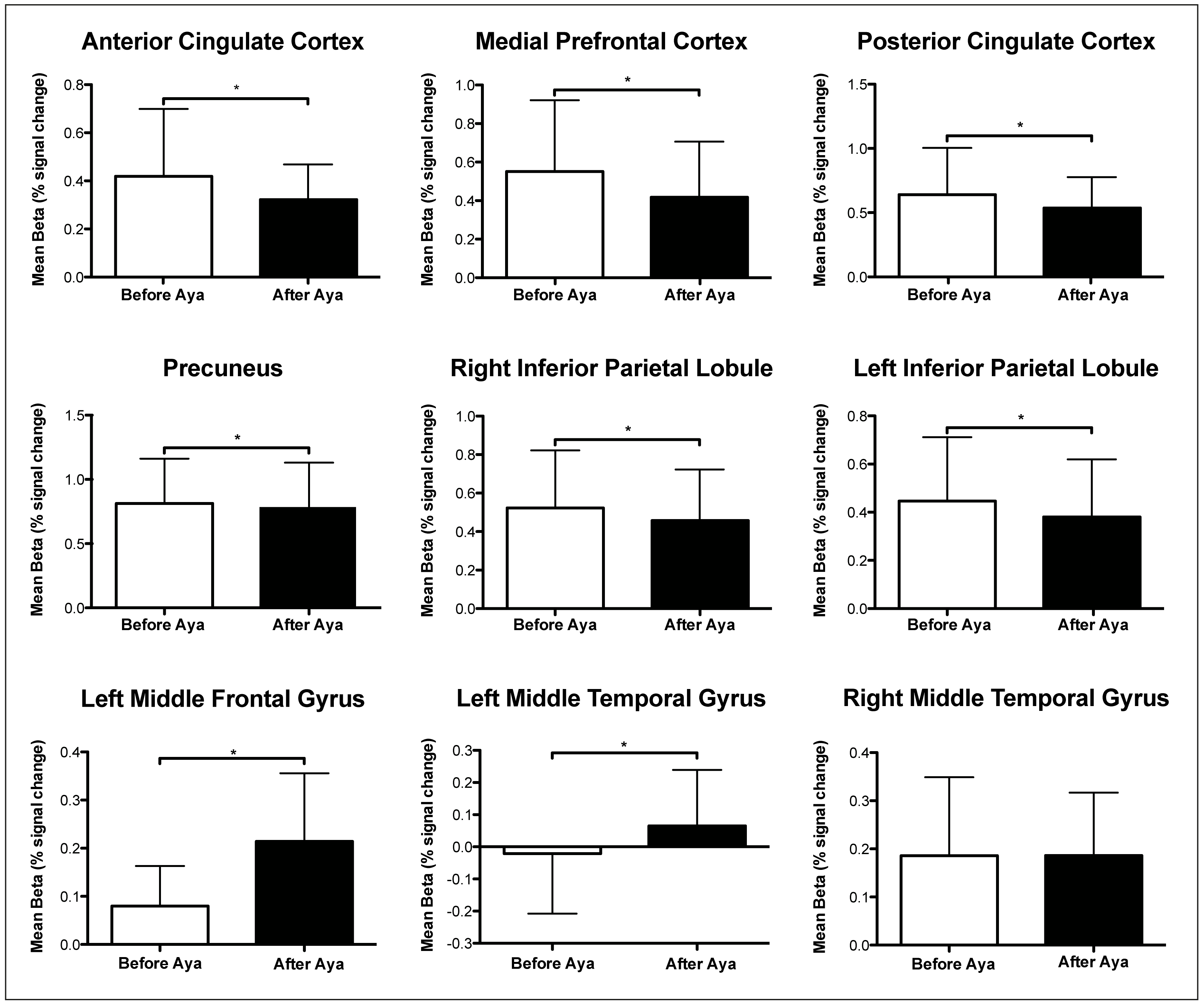

Supplement: S3 Fig — Bar plots of the mean β-values from ROIs of the DMN where BOLD signal was altered after Ayahuasca ingestion. Decreases were found in most of ROI: ACC, MPFC, PCC, PC and bilateral IPL. *p < 0.01 correct for multiple comparisons by the number of ROI (nine). (TIF) [file pone.0118143.s003.tif]

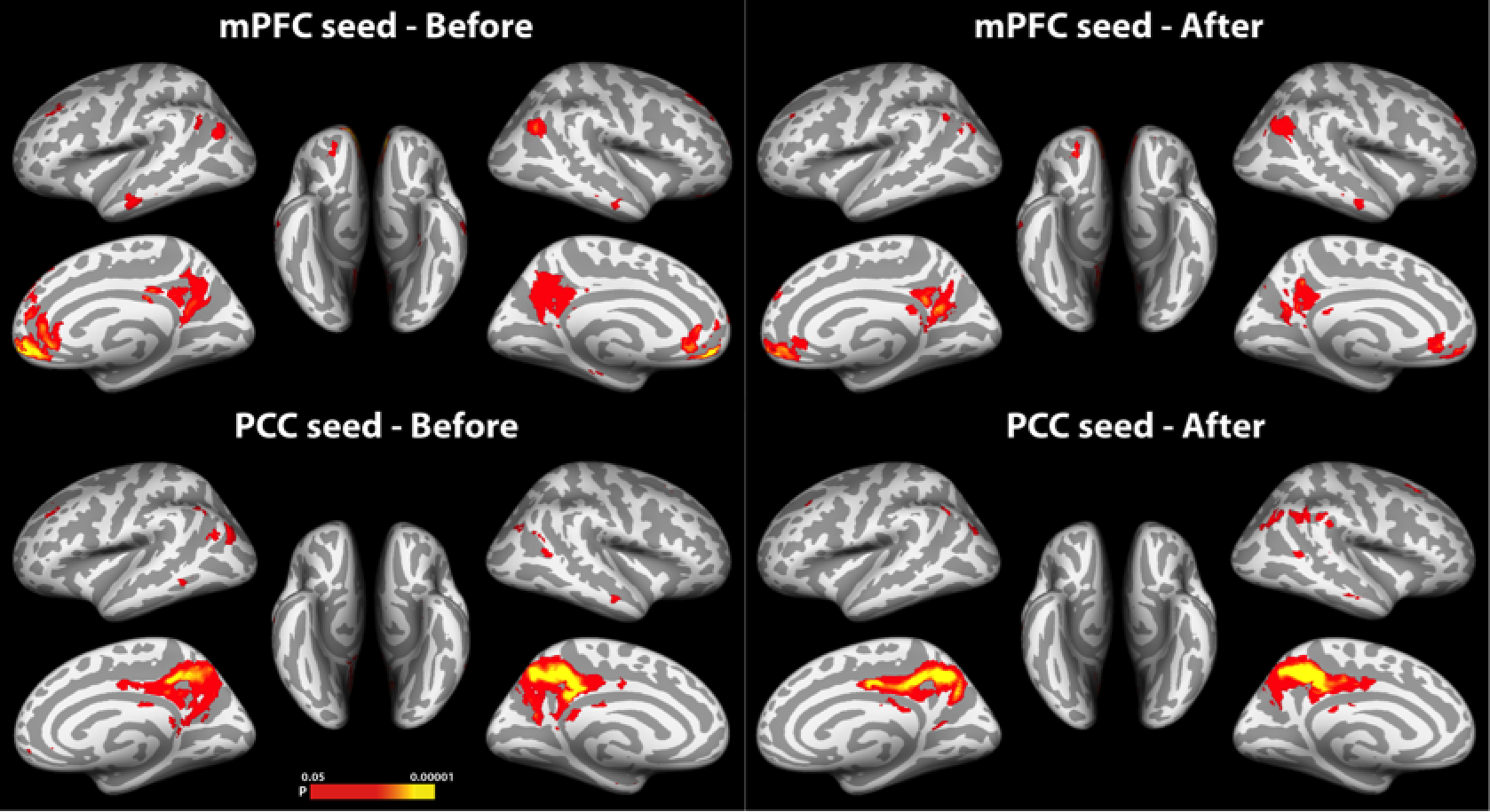

Supplement: S4 Fig — Maps are shown at the two moments: before (left side) and after (right side) Ayahuasca ingestion. The resulting statistical maps were thresholded at p < 0.05 uncorrected. (TIF) [file pone.0118143.s004.tif]

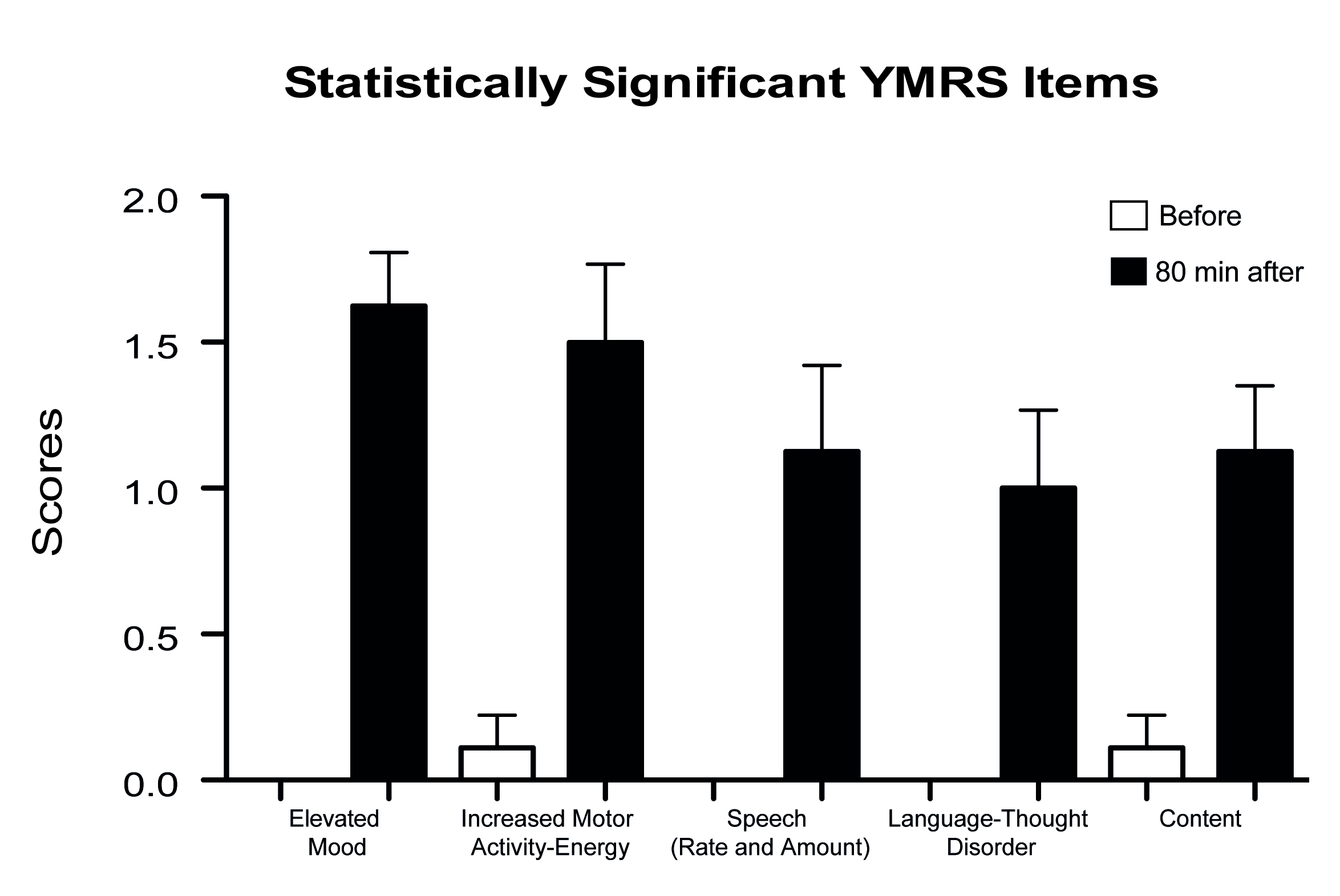

Supplement: S5 Fig — Only 5 out of the 11 YMRS items showed statistically significant changes after Ayahuasca intake. Bars present score values (mean + SE). P < 0.05 uncorrected. (TIF) [file pone.0118143.s005.tif]
